# Tabu search for a parallel-machine scheduling problem with periodic maintenance, job rejection and weighted sum of completion times

**DOI:** 10.1007/s10951-021-00711-9

**Published:** 2021-10-17

**Authors:** Hanane Krim, Nicolas Zufferey, Jean-Yves Potvin, Rachid Benmansour, David Duvivier

**Affiliations:** 1 Polytechnic University of Hauts-de-France LAMIH, UMR CNRS 8201, 59313 Valenciennes Cedex 9, France; 2 Geneva School of Economics and Management, GSEM, University of Geneva, 1211 Geneva 4, Geneva, Switzerland; 3 Department of Computer Science and Operations Research, University of Montreal, P.O.Box 6128, Montreal, H3C 3J7 Canada; 4 INSEA, SI2M Laboratory, Rabat, Morocco; 5 Interuniversity Research Centre on Enterprise Networks, Logistics and Transportation (CIRRELT), Montreal, Canada

**Keywords:** Parallel machine scheduling, Job rejection, Periodic maintenance, Mixed-integer-linear-program, Metaheuristic, Tabu search, Lexicographic optimization

## Abstract

We consider in this work a bicriteria scheduling problem on two different parallel machines with a periodic preventive maintenance policy. The two objectives considered involve minimization of job rejection costs and weighted sum of completion times. They are handled through a lexicographic approach, due to a natural hierarchy among the two objectives in the applications considered. The main contributions of this paper are first to present a new problem relevant to practice, second, to develop a mixed-integer-linear-program model for the problem, and third, to introduce two generalizable tabu-search metaheuristics relying on different neighborhood structures and solution spaces. Computational results for 120 instances (generated from a real case) are reported to empirically demonstrate the effectiveness of the proposed metaheuristics.

## Introduction

Scheduling problems have been extensively studied in the literature under the assumption that all jobs have to be processed. However, in many practical cases, one may wish or may be forced to postpone the processing of some jobs, although at some cost. Accordingly, a decision has to be made about jobs that will be accepted and those that will be rejected to produce a good schedule. Nowadays, this situation is observed in several companies with a weekly planning (e.g., pharmaceutical products, luxury watches, fast moving consumer goods). Typically, rejected jobs will get a larger weight or priority the next week. At the same time, the parallel-machine scheduling problem has been extensively studied due to its practical applications in various manufacturing systems such as printed circuit board manufacturing, group technology cells and injection molding processes. However, few studies have been done in the context of parallel-machine scheduling with job rejection.

Maintenance is another aspect closely connected to production scheduling in real manufacturing settings. One of the most common assumptions in the scheduling literature is that the machines or processors are always available, but, in practice, they may have to be stopped due to failures or preventive maintenance (PM). In particular, the importance of PM has been gradually recognized by decision makers as a mean to avoid machine failures. PM is performed when the machines are idle and, consequently, represents a source of machine unavailability. Trade-offs to be found between PM and production activities have led researchers to investigate different ways of jointly scheduling both activities. Production is expected to be more efficient and revenues to increase when PM is well managed.

In this regard, we address a scheduling problem (P) with two different parallel machines (it is formally a *2-Parallel Machines problem with Periodic Maintenance, Job Rejection and Weighted sum of Completion Times*). In this problem, the two machines must undergo periodic preventive maintenance over the scheduling horizon. The two machines are different as PMs must be done more frequently on the second machine. Solution quality is measured with two criteria. The first one is the total cost of rejected jobs, and the second one is the weighted sum of job completion times. In the former case, jobs are rejected when the capacity of both machines is reached. In the latter case, the weights can stand for the holding or inventory cost of the corresponding jobs as well as their priority level (importance, urgency). A strategy based on lexicographic optimization (LO) is proposed to deal with this multiobjective problem. In LO, the decision maker establishes beforehand a priority order among the optimization objectives, where each higher-level objective is infinitely more important than any lower-level objective. LO is a convenient approach to address multiobjective problems in practice, as reported in Zykina (2004), Ehrgott (2005), Thevenin et al. (2017b), Prats et al. (2010), Solnon et al. (2008), T’kindt and Billaut (2006)).

The contributions of this paper are the following: (1) we propose a new problem relevant to practice; (2) we formulate the problem with a Mixed Integer Linear Program (MILP); (3) a diversified panel of solutions methods is proposed, namely a greedy constructive procedure, two tabu-search approaches relying on various neighborhood structures and different solution spaces, and a baseline local-search heuristic aimed at representing a current-practice rule. The solution methods are easily generalizable for various job-scheduling contexts. (4) We generate 120 instances derived from an industrial case, considering up to 330 jobs. (5) We compare all the methods not only with respect to the two considered objective functions, but also with respect to the number of rejected jobs, which is an important key performance indicator (KPI) for practitioners.

The remainder of this paper is organized as follows. Section 2 presents the literature review dealing with order acceptance and scheduling, job rejection, periodic maintenance and multi-availability constraints. Section 3 proposes a MILP for the problem. The greedy constructive heuristic is developed in Sect. 4. Two tabu-search metaheuristics and a baseline local search heuristic are designed in Sect. 5. Section 6 reports computational results. Finally, Sect. 7 ends the paper with a conclusion and some perspectives for the future.

## Literature review

Based on the three-field notation $\alpha \mid \beta \mid \gamma$ known as the Graham triplet (Graham et al. 1979), our problem (P) can be denoted as $P2 \mid pm \mid \sum _{j=1}^{n}u_j,\sum _{j=1}^{n} w_j C_{j}$. The first field ($\alpha$) means that there are two parallel machines. The second field ($\beta$) indicates that a periodic preventive maintenance (*pm*) must be performed on each machine. Finally, the last field ($\gamma$) represents the objective functions (see the notation used in Sect. 3). To the best of our knowledge, problem (P) has never been studied in the literature. Nonetheless, Sects. 2.1 to 2.4 will review works that are related to this problem. Sections 2.1 and 2.2 are dedicated to the order acceptance and scheduling literature and to the scheduling problem with job rejection literature, respectively. In both cases, the same problematic issue is addressed, namely job scheduling when the production capacity is not sufficient to allow all jobs to be scheduled. This situation leads to the rejection (resp. acceptance) of some of them, which is penalized (resp. rewarded) in the objective function. Sections 2.3 and 2.4 focus on the maintenance and on the lexicographic optimization aspects in the context of job scheduling. We conclude with Sect. 2.5 by motivating our methodological choices with respect to the literature.

### Order acceptance and scheduling

A taxonomy and a general review on order acceptance and scheduling (OAS) can be found in Slotnick (2011). This problem is to jointly decide about job acceptance and the scheduling of accepted jobs. Different problem characteristics and problem-solving methodologies, starting from this basic scheme, have been proposed in the literature. In the following, papers dealing with a single machine and different objective functions are reviewed, followed by a discussion on problems with two or more machines.

#### Single machine

Oğuz et al. (2010) consider the single-machine scheduling problem where job acceptance depends on the release date, due date, deadline, processing time, sequence-dependent setup time and revenue. The main objective is the maximization of the total revenue. The authors propose a MILP and also develop three heuristic algorithms to solve their problem. Based on the same objective function, Cesaret et al. (2012) propose a tabu search to solve a problem that considers sequence-dependent setup times and tardiness penalties. Nobibon and Leus (2011) generalize two existing problems defined in a single-machine environment, that is, the order acceptance and scheduling problem with weighted-tardiness penalties reported in Slotnick and Morton (2007) and the total weighted tardiness scheduling problem reported in Potts and Van Wassenhove (1985). The generalized problem reduces to the latter when the pool of firm planned orders is empty and all jobs can potentially be rejected. To solve their generalized problem, the authors propose a MILP and two exact branch-and-bound algorithms. They report solving instances with up to 50 jobs in less than 2 h. In Thevenin et al. (2016), the authors address a production scheduling problem in a single-machine environment with earliness and tardiness penalties, sequence-dependent setup times and costs. The objective function includes setup costs, job rejection penalties and weighted tardiness penalties. The authors propose various methods to solve this problem, ranging from a basic greedy algorithm to sophisticated metaheuristics (e.g., tabu search, adaptive memory algorithm). In another work by the same authors Thevenin et al. (2015), sequence-dependent setup times and setup costs between jobs of different families, release dates, deadlines and job rejection are taken into account. They propose and compare a constructive heuristic, local search methods, and population-based algorithms. Recent papers dealing with OAS in a single-machine environment take into account machine availability constraints, as in Zhong et al. (2014). Here, the authors propose a pseudo-polynomial algorithm for fixed time intervals between two consecutive PMs.

#### Multiple machines

In Ou and Zhong (2017), the authors study the OAS problem for *n* jobs on *m* parallel machines where the number of rejected jobs should not exceed a given limit *L*. The objective is to minimize the completion time of the last scheduled job plus the total cost of rejected jobs. For the special case of a single machine, they present an exact algorithm of complexity $O(n \cdot log(n))$. For *m* machines, they first propose a heuristic of complexity $O(n \cdot log(n))$ with a worst-case bound of $2 - \frac{1}{m}$. They also develop a heuristic based on LP-relaxation and bin-packing techniques. The OAS with two machines in a flow shop is considered in Wang et al. (2013). The authors present a heuristic and a branch-and-bound algorithm based on dominance rules and relaxation techniques. Their objective is to maximize the total net profit of accepted jobs, where the latter is the revenue minus the weighted tardiness. In Wang et al. (2015), the authors solve a scheduling problem with two parallel machines with two heuristics and an exact algorithm, using some properties of optimal solutions to maximize the total profit. In another environment with parallel machines, Jiang et al. (2017) study the OAS problem with batch delivery in a supply chain consisting of a manufacturer and a customer. The objective is to minimize the weighted sum of the maximum lead times of accepted jobs and the total delivery cost. To solve the problem, two approximation algorithms are proposed. Finally, Emami et al. (2016) report a MILP model and a Lagrangian relaxation algorithm to solve an OAS problem with the objective of maximizing the total profit.

### Scheduling problem with job rejection

The scheduling problem with job rejection has been studied in different contexts, as indicated in a recent survey (Shabtay et al. 2013), and is motivated by industrial applications (Thevenin et al. 2017a), although mostly for single-machine problems.

In Li and Chen (2017), the authors consider the scheduling problem with job rejection and a maintenance activity that becomes less effective over time. The main objective is to determine the timing of the maintenance activity and the sequence of accepted jobs to minimize the scheduling cost of accepted jobs plus the total cost of rejected jobs. The authors provide polynomial time algorithms for this problem. Shabtay et al. (2012) propose a bicriteria analysis of a large class of single-machine scheduling problems with a common property, namely the consideration of rejection costs plus other additional criteria (makespan, sum and variation of completion times, earliness and tardiness costs).

Since scheduling with rejection is mostly studied in bicriteria contexts (Shabtay et al. 2013), concepts from the theory of bicriteria scheduling are commonly used when dealing with such problems. Below, we review papers addressing the weighted sum of completion times and the total cost of rejected jobs. Cao et al. (2006) first prove that the problem for a single machine is NP-hard. A few years later, a pseudo-polynomial algorithm and a fully polynomial time approximation scheme (FPTAS) for multiple parallel machines are proposed by Zhang et al. (2009). Engels et al. (2003) also report more general techniques such as linear programming relaxations. In Moghaddam et al. (2012), the authors study a single-machine scheduling problem with job rejection, while considering again minimization of the weighted sum of completion times plus the total cost of rejected jobs. They propose a mathematical formulation and three different bi-objective simulated annealing algorithms to estimate the Pareto-optimal front for large-size instances. The authors in Zhong et al. (2017) study a scheduling problem on two parallel machines with release times and job rejection. The objective is to minimize the makespan of accepted jobs plus the total cost of rejected jobs. They develop a $(1.5 + \varepsilon )$-approximation algorithm to solve the problem. Ou et al. (2015) consider *m* parallel machines in a context where job rejection is allowed. The objective is to minimize the makespan plus the total cost of rejected jobs. They develop a heuristic of complexity $O(n \cdot log(n) + n/\varepsilon )$ to solve the problem with a worst-case bound of $1.5 + \varepsilon$. With the same goal, Zhong and Ou (2017) present a 2-approximation algorithm with a complexity of $O(n \cdot log(n))$ by making use of specific data structures. The authors also propose a PTAS to solve the problem. In Ma and Yuan (2016), the authors consider that the information about each job, including processing time, release date, weight and rejection cost, is not known in advance. They develop a technique named Greedy-Interval-Rejection to produce good solutions. Finally, the authors in Agnetis and Mosheiov (2017) consider the minimization of the makespan in a flow shop with position-dependent job processing times and job rejection. A polynomial time procedure is proposed to solve this problem.

### Periodic maintenance and multi-availability constraints

The authors in Kaabi and Harrath (2014) have written a comprehensive survey about scheduling in parallel-machine environments in the presence of availability constraints (which can be induced, in particular, by maintenance activities). Sun and Li (2010) consider two problems. In the first problem, they minimize the makespan on two parallel machines when maintenance activities are performed periodically. In the second problem, maintenance activities are determined jointly with job scheduling, while minimizing the sum of the job completion times. They introduce an algorithm of complexity $O(n^{2})$ and show that the classical shortest processing time algorithm (SPT) is efficient for the second problem with a worst-case bound less than or equal to $1+2 \cdot \sigma$, where $\sigma =t/T$, and *T* is the maximum continuous working time for each machine and *t* is the time required to perform each maintenance activity. Li et al. (2017) investigate a parallel-machine scheduling problem where each machine must undergo periodic maintenance. The authors propose two mathematical programming models and two heuristic approaches to address instances of large size. In Qi et al. (2015), the authors investigate a scheduling problem on a single machine with maintenance, in which the starting time of the maintenance is given in advance but its duration depends on the previous machine load.

### Multiobjective scheduling problem using lexicographic optimization

LO is particularly relevant for industrial applications, as highlighted by Gallay and Zufferey (2018). LO is widely used in control engineering and scheduling applications (T’kindt and Billaut 2006; Aggelogiannaki and Sarimveis 2006; Kerrigan and Maciejowski 2002; Ocampo-Martinez et al. 2008; Respen et al. 2016). In a work closely related to ours, the authors in Thevenin et al. (2017b) model a parallel-machine scheduling problem with job incompatibility through an extension of the graph coloring problem. Different objectives like makespan, number of job preemptions and total time spent by the jobs in the production shop are considered and addressed through LO. A mathematical model, two greedy constructive algorithms, two tabu search methods and an adaptive memory algorithm are proposed to solve the problem.

### Motivation of our methodological choices with respect to the literature

In this subsection, we highlight how the literature led us to consider the proposed methods, neighborhood structures and objective-function priority.

As discussed before, despite the industrial relevance of the combination of features that characterizes the considered problem (P) (e.g., different parallel machines, rejection costs, inventory penalties, maintenances), it has not attracted attention in academia. Relying on the above literature review, we can, however, deduce that the following ingredients are relevant when facing the features of (P): small-sized instances can be solved with the use of a MILP formulation, whereas meta/heuristics are required for large-sized instances. For tackling the large-sized instances, constructive heuristics are useful, in particular for generating initial solutions for local-search algorithms. Our motivation to employ tabu search comes from its great success for various job-scheduling problems, in particular in a parallel-machine production environment when various objectives are considered (Respen et al. 2016; Thevenin et al. 2017a, b).

Regarding the neighborhood structures, four moves are widely employed and well known in the job-scheduling literature (Thevenin et al. 2015): insert (i.e., move a job somewhere else in the schedule), swap (i.e., exchange two jobs in the schedule), drop (i.e., remove a job from the schedule) and add (i.e., insert a non-scheduled job in the schedule). None of these neighborhood structures can be used alone (in particular when considering job rejection), as a single type of move does not allow to reach all the solutions of the solution space (which means that the search space would not be connected). Several papers [e.g., Shin et al. (2002)] confirmed that using jointly several types of moves leads to better results. For these reasons, the local search that we propose employs various types of moves, including swapping a scheduled job with a non-scheduled jobs (which corresponds to a drop-and-add combination) or reinserting a block of jobs somewhere else in the schedule (which corresponds to an imposed sequence of insert moves).

**Fig. 1:**
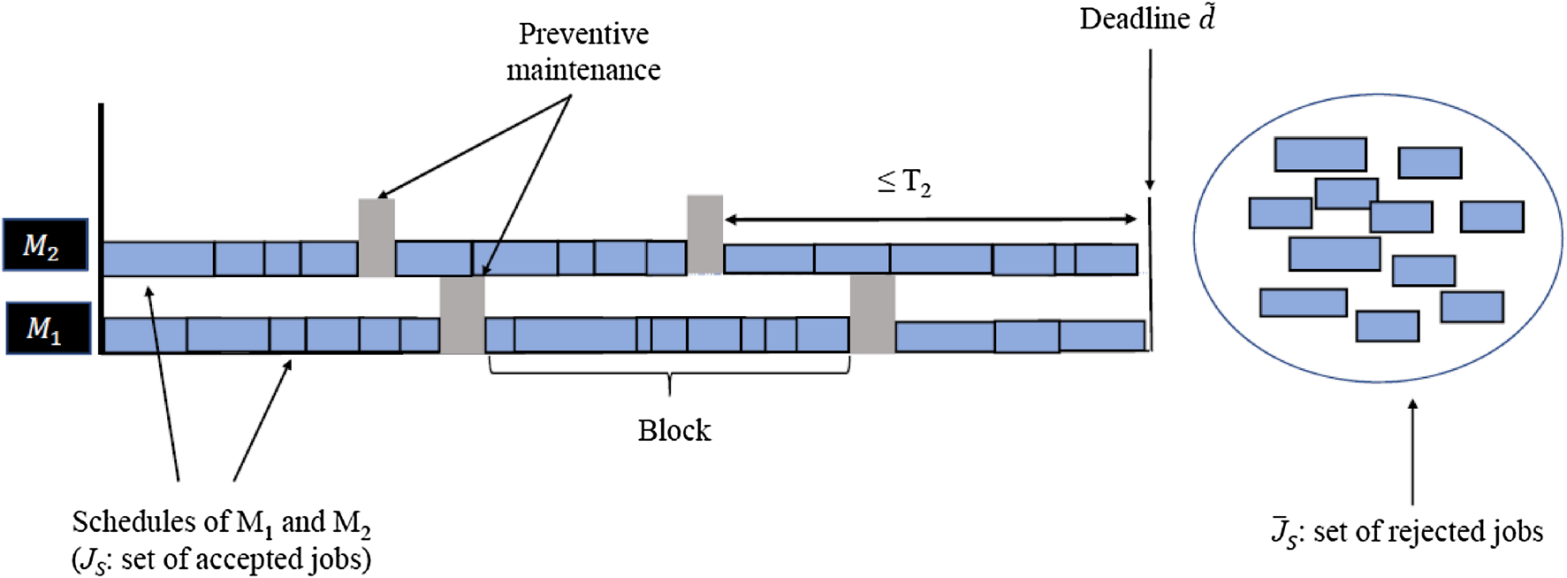
feasible solution of problem (P)

The two objective functions $f_1$ (rejection penalty, which is related to shortage costs) and $f_2$ (weighted sum of completion times, which is related to inventory costs) are well known in the literature. However, the consideration of both of them, furthermore in a lexicographic fashion (i.e., $f_1 > f_2$), is new. Their joint consideration within such a hierarchy corresponds to natural priorities encountered in practice, for example Respen et al. (2016), where minimizing shortage is more important than minimizing inventory penalties, Thevenin et al. (2018), where maximizing the gain of the scheduled jobs is more important than minimizing an inventory-oriented penalty, and more generally, throughout the order-acceptance-and-scheduling literature (Cesaret et al. 2012; Shabtay et al. 2013).

## Mathematical model

In the following, we first introduce some notation and a brief description of our problem. This is followed by the MILP.

### Formal description of problem (P)

Let *J* be a set of *n* independent jobs to be scheduled on two parallel, different machines $M_i$, $i \in I = \{1, 2\}$. The planning horizon is five days (i.e., 7200 min). Accordingly, we define ${\tilde{d}}$ = 7200 min as the common deadline for all jobs in set *J*. If a job cannot be feasibly scheduled during the current week, it is then postponed to the next week and a rejection cost is incurred. A feasible solution *S* of problem (P) is illustrated in Fig. 1. It is made of two schedules on machines $M_1$ and $M_2$, with the corresponding sets $J_S$ and ${\overline{J}}_S$ of accepted and rejected jobs, respectively. Each machine $M_i$ must undergo a PM at intervals that cannot exceed $T_i$ minutes. In other words, the interval between the end time of a given PM and the start time of the next PM cannot exceed $T_i$ minutes. The jobs scheduled between two consecutive PMs define a block, where $B_{k}^{i}$ is the *k*th block on machine $M_i$ scheduled between the $(k-1)$th and *k*th PMs (the 0th and last PMs are the start and end of the schedule, respectively). The scheduling of a PM activity on each machine $M_i$ is flexible and can actually occur before $T_i$ minutes have elapsed, if it is not possible to avoid it or if it is beneficial to do so. Accordingly, the time length of a block is variable, although it can never exceed $T_i$ for machine $M_i$. The duration of a PM activity on machine $M_i$ is denoted by $\delta _i$. As illustrated in Fig. 1, there is no idle time in a schedule between two consecutive jobs or between a job and a PM.

Each job $j \in J$ is characterized by a known processing time $p_j$, a rejection cost $u_j$ and a weight $w_j=h_j+b_j$ which is the sum of its inventory penalty $h_j$ and its priority level $b_j$, where a larger $b_j$ corresponds to a larger penalty. It should also be noted that no preemption is allowed. The two machines are different in the sense that PMs must be done more frequently on $M_2$. Thus, the maximum time interval $T_2$ between two consecutive PMs is smaller than $T_1$ (i.e., $T_2 < T_1$). A feasible solution *S* of problem (P) is evaluated first through objective $f_1$, which is the total rejection cost of the jobs in ${\overline{J}}_S$, and second through objective $f_2$, which is the weighted sum of the completion times of the jobs in $J_S$. Formally, we have $f_2 = \sum _{j=1}^{n}w_j C_j$, where $C_j$ is the completion time of job *j*. With respect to $f_2$, the WSPT (weighted shortest processing time) rule introduced in Smith (1956) is particularly important, because it optimally solves the $1 \mid \mid \sum _{j=1}^{n}w_{j}C_{j}$ scheduling problem, which minimizes the weighted sum of completion times on a single machine without side constraints. The WSPT rule states that the jobs should be scheduled in decreasing order of the $w_j/p_j$ ratios. This rule will be exploited in our algorithms, although in a heuristic way since we have two machines with some operational constraints.

In our study and in line with the literature (Thevenin and Zufferey 2019), $u_j$ depends on $b_j$ and $p_j$. Moreover, we have calibrated $f_2$ in order to give the same importance to the inventory penalty $h_j$ and to the priority $b_j$ of each job *j*, as both values belong to the same interval (which is [10, 30] in our experiments). Note that if the two components $b_j$ and $h_j$ of $w_j = b_j + h_j$ are fully in conflict for a given job *j*, it means that $h_j = 10$ (or 30, respectively) whereas $b_j = 30$ (or 10, respectively). As a result, $w_j = b_j + h_j = 40$, which corresponds to the average value of $w_j$ over all jobs. In other words, a job *j* such that its inventory penalty and its priority are fully in conflict will have a medium weight (or importance $w_j$) in the scheduling process, which is consistent with $f_2$. In contrast, if $b_j$ and $h_j$ are not in conflict, it means either that $w_j$ is low (typically below 30) or high (typically above 50). Therefore, from this calibration of $(f_2, u_j, b_j, h_j)$, we can deduce the following hierarchy of KPIs: (1) minimize the rejection costs; (2) minimize the bad scheduling of high-priority jobs; (3) minimize the inventory penalties. This hierarchy is in line with the industrial KPIs of various companies (Thevenin et al. 2015, 2016; Respen et al. 2017; Thevenin and Zufferey 2019). Note, however, that our models and methods proposed below are general: they do not depend on the calibration of $(f_2, u_j, b_j, h_j)$. Actually, this calibration has to be made in collaboration with the involved industrial partner in order to capture its KPIs.

### MILP for problem (P)

The mathematical programming formulation of problem (P) is presented below. It involves five different types of decision variables.

$$\begin{aligned}&C_j : \text{ completion } \text{ time } \text{ of } \text{ job } j \\&m^i_{k} : \hbox { start time of PM} k \hbox {on machine} M_i \\&x_{lj}^{i} = {\left\{ \begin{array}{ll} 1 &{} \hbox { if job}\ l \hbox { is scheduled before job }j \hbox { on machine }M_i\\ 0 &{} \text {otherwise} \end{array}\right. } \\&z_{j}^{i} = {\left\{ \begin{array}{ll} 1 &{} \hbox { if job} j \hbox { is scheduled on machine} M_i \\ 0 &{} \text {otherwise} \end{array}\right. } \\&y_{jk}^{i} = {\left\{ \begin{array}{ll} 1 &{} \hbox {if job } j \hbox { is scheduled in block} k \hbox { on machine }M_i \\ 0 &{} \text {otherwise} \end{array}\right. } \end{aligned}$$

It is important to note that job *j* is rejected when $z_{j}^{1} = z_{j}^{2} = 0$. For the sake of the MILP formulation, two dummy jobs 0 and $n+1$ with no processing time are added to the model with completion times $C_0 = 0$ and $C_{n+1} = {\tilde{d}}$. We also have $m^1_{0} = m^2_{0} = 0$. Note finally that *M* is an arbitrary large number.

Due to the lexicographic ordering of the two objectives, problem (P) can be solved optimally in two steps with an exact solver. A first model is solved with the objective of minimizing $f_1$ (while ignoring $f_2$). Next, a second model is solved, with the objective of minimizing $f_2$, that includes a constraint for not exceeding the optimal value of $f_1$.

The first model is the following:

1 $$\begin{aligned}&\min \, (f_1)= \displaystyle \sum _{j=1}^{n}u_j(1-(z_j^{1}+z_j^{2})) \end{aligned}$$

2 $$\begin{aligned}&m_{k}^{i} \le m_{k-1}^{i} + \delta _{i} + T_{i} \quad \forall i \in I, \, \forall k \in J {}\end{aligned}$$

3 $$\begin{aligned}&m_{k}^{i} + \delta _i\le {\tilde{d}}\quad \forall i\in I, \, \forall k \in J {} \end{aligned}$$

4 $$\begin{aligned}&C_j\le m_k^{i}+M(1-y_{jk}^{i}) \quad \forall i\in I, \forall j, k \in J {}\end{aligned}$$

5 $$\begin{aligned}&C_j\ge m_{k-1}^{i}+\delta _{i}+p_{j}y_{jk}^{i}-M(1-y_{jk}^{i}) \quad \forall i\in I, \, \nonumber \\&\quad \forall j, k \in J {} \end{aligned}$$

6 $$\begin{aligned}&\displaystyle \sum _{l=1, l \ne j}^{n+1} x_{jl}^{i} = z_{j}^{i} \quad \forall i\in I, \, j = 0,\ldots ,n { }\end{aligned}$$

7 $$\begin{aligned}&\displaystyle \sum _{l=0, l\ne j}^{n} x_{lj}^{i} = z_{j}^{i} \quad \forall i\in I, \, j = 1,\ldots ,n+1 { }\end{aligned}$$

8 $$\begin{aligned}&x_{lj}^{1} + x_{lj}^{2} \le 1 l = 0,\ldots , n, \ j = 1,\ldots n+1, \, l \ne j \end{aligned}$$

9 $$\begin{aligned}&C_j \ge p_j(z_{j}^{1}+z_{j}^{2}) \quad \forall j \in J \end{aligned}$$

10 $$\begin{aligned}&C_j \le {\tilde{d}}(z_{j}^{1}+z_{j}^{2}) \quad \forall j \in J \end{aligned}$$

11 $$\begin{aligned}&C_l \le C_j-p_jx_{lj}^{i}+ {\tilde{d}}(1-x_{lj}^{i}) \quad \forall i\in I, \, l = 0,\ldots ,n, \nonumber \\&\quad j = 1,\ldots ,n+1, \, l \ne j { }\end{aligned}$$

12 $$\begin{aligned}&\displaystyle \sum _{j=1}^{n} p_{j}y_{jk}^{i} \le T_i \quad \forall i\in I, \, \quad \forall k \in J \end{aligned}$$

13 $$\begin{aligned}&\displaystyle \sum _{k=1}^{n} y_{jk}^{i} = z_{j}^{i} \quad \forall i \in I, \, \quad \forall j \in J { }\end{aligned}$$

14 $$\begin{aligned}&y_{jk}^{1}+y_{jk}^{2}\le 1 \quad \forall j, k \in J { }\end{aligned}$$

15 $$\begin{aligned}&z_{j}^{1}+z_{j}^{2} \le 1 \quad \forall j \in J { }\end{aligned}$$

16 $$\begin{aligned}&z_{l}^{i}+z_{j}^{i} \ge 2(x_{lj}^{i}+x_{jl}^{i}) \quad \forall i \in I, \, \quad \forall l, j \in J, \ l \ne j { }\end{aligned}$$

17 $$\begin{aligned}&C_0 = 0, \, C_{n+1} = {\tilde{d}},\, m_0^{1} = m_0^{2} = 0 \end{aligned}$$

18 $$\begin{aligned}&y_{jk}^{i} \in \lbrace 0,1\rbrace \quad \forall i \in I, \, \forall j, k \in J {}\end{aligned}$$

19 $$\begin{aligned}&z_{j}^{i} \in \lbrace 0,1\rbrace \quad \forall i \in I, \, j = 0,\ldots ,n+1 {}\end{aligned}$$

20 $$\begin{aligned}&x_{lj}^{i} \in \lbrace 0,1\rbrace \quad \forall i \in I, \, l = 0,\ldots ,n, \ \nonumber \\&\quad j = 1,\ldots ,n+1, \ l \ne j { }\end{aligned}$$

21 $$\begin{aligned}&m_{k}^{i} \ge 0 \quad \forall i \in I, \, \forall k \in J { } \end{aligned}$$

This model relies on the fact that the number of PMs and blocks on a machine is at most the total number of jobs. Since a machine has normally fewer PMs and blocks in a solution, there are variables $m^i_{k}$ that do not correspond to real PMs and whose values do not matter. Equation (1) corresponds to the first objective function considered in this work. Constraints (2) define the relationship between the start time of two consecutive PMs on a machine. The end time of a PM cannot exceed the due date ${\tilde{d}}$, as indicated by constraints (3). Constraints (4) and (5) establish a relationship between variables $C_j$ and $m_i^k$. Basically, they state that the completion time of a job assigned to block *k* on a machine lies between the end time of PM $k-1$, plus the processing time of the job, and the start time of PM *k*. Constraints (6) indicate that every scheduled job, including job 0, must have a successor. Constraints (7) indicate that every scheduled job, including job $n+1$, must have a predecessor. Constraints (8) state that two jobs scheduled consecutively must be assigned to the same machine. Constraints (9) and (10) define bounds on the completion time of each scheduled job. In particular, they force the completion time of a rejected job to be 0. Constraints (11) indicate that two jobs scheduled on the same machine cannot overlap. Constraints (12) state that the sum of processing times over all jobs in the same block on machine $M_i$ must be less than or equal to $T_i$. Constraints (13) indicate that an accepted job must be part of a block on a machine. Constraints (14) force each accepted job to be scheduled in a block of either machine $M_1$ or $M_2$ but not both. Similarly, constraints (15) force each accepted job to be scheduled either on $M_1$ or $M_2$ but not both. Constraints (16) state that if two jobs *l* and *j* are scheduled on the same machine, then *l* is scheduled either before or after *j*. Constraints (17) set the completion times of dummy jobs 0 and $n+1$, and the start time of the dummy PM 0 on each machine. Constraints (18), (19) and (20) define the binary variables, whereas the continuous variables are defined in constraints (21).

Let $f_1^{\star }$ be the optimal value of $f_1$ after solving the above model. In a second step, constraint $f_1\le f_1^{\star }$ is added to the model and the latter is solved with objective $f_2$ only. In other words, the model below is considered:

22 $$\begin{aligned}&\min \, (f_2)=&\displaystyle \sum _{j=1}^{n} w_jC_j \end{aligned}$$

23 $$\begin{aligned}&s.t.&\text {Constraints } (2-20) \end{aligned}$$

24 $$\begin{aligned}&\displaystyle \sum _{j=1}^{n}u_j(1-(z_j^{1}+z_j^{2})) \le f_1^{\star } \end{aligned}$$

Equation (22) corresponds to the second objective, while constraint (24) bounds the value of the first objective. The solution obtained at the end of this second step is the optimal solution of (P). We observed that the CPLEX solver could only be used for small instances. More precisely, we were able to solve instances with up to 25 jobs within approximately 16 h of computation time. But CPLEX had to be stopped after 24 h of computation time, with a very large optimality gap, on instances with 40 jobs. These results support the use of heuristics and metaheuristics for instances of larger, more realistic, size.

In the following, our problem-solving methodologies are presented, starting with the greedy heuristic to generate a first feasible schedule, which is then improved with tabu search-based metaheuristics.

## Greedy heuristic *GrH*

The greedy heuristic *GrH* calls a construction procedure which is aimed at producing a feasible schedule of good quality from a given set of jobs. In particular, *GrH* calls the construction procedure within a loop where the considered set of jobs is gradually reduced until all jobs in the reduced set can be scheduled. In each proposed procedure of this work, ties are broken randomly if no other information is provided.

### Main procedure

We can see from the description in Algorithm 1 that *GrH* starts by calling the greedy construction procedure (presented in Algorithm 2) with a set of jobs $J'$, which is initially the set of all jobs *J* (steps 1 and 2). The construction procedure then returns a feasible solution *S*, which is associated with a set of accepted jobs $J_S$ and a set of rejected jobs ${\overline{J}}_S$. If not all jobs in $J'$ are accepted in solution *S*, we select the $|J_S|$ jobs in *J* with the largest $u_j$ to obtain a smaller set $J'$ (step 3a). The construction procedure is then called again with the new $J'$ (step 3b). If the solution *S* obtained does not contain all jobs in $J'$, we select again the $|J_S|$ jobs in *J* with the largest $u_j$ to obtain a new set $J'$ (step 3a again), and the construction procedure is called with the latter (step 3b again). This is repeated until all jobs in $J'$ are accepted in the obtained solution *S*, that is, when $J_S = J'$. Thus, the aim of the loop (step 3) is to schedule as many jobs as possible with the largest rejection costs, since $f_1$ is the main objective. Next (step 4), we consider the rejected jobs in the last solution obtained and we try to add them at the end of the schedule of machines $M_1$ and $M_2$. These jobs are considered one by one in decreasing order of rejection costs. First, we check if the current job *j* can be added without exceeding the deadline ${\tilde{d}}$ (if the addition of job *j* leads to exceeding the due time of the next PM, a PM must also be added before job *j*). If job *j* is feasible on a single machine, it is added to this machine; if job *j* is feasible on both machines, it is added to the machine with minimum completion time $C_j$ (in order to account for $f_2$); if job *j* is not feasible on any machine, it is skipped.

One can remark that steps 3 and 4 are complementary. Indeed, step 3 aims at scheduling as many jobs with the top (i.e., highest) rejection costs. Next, step 4 fills the “holes” in the schedule, by greedily adding other jobs to the solution (i.e., with respect to decreasing rejection costs). 
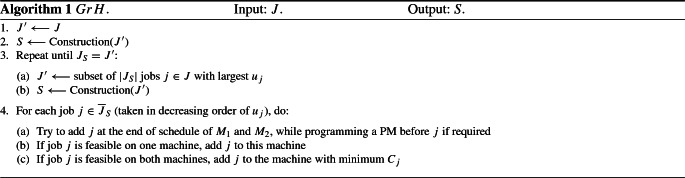


### Construction procedure

The construction procedure, described in Algorithm 2, produces a solution *S* from scratch in a greedy way. Using the set $J'$ of jobs provided in input, a new job *j* is selected and added, at each iteration, at the end of the schedule of $M_1$ or $M_2$. This is repeated (step 3) as long as there are jobs which can be added to the schedule of at least one machine without exceeding the deadline ${\tilde{d}}$ (if the addition of job *j* leads to exceeding the due time of the next PM, a PM must also be added before job *j*). This feasibility check is performed through calls to AssignFeasible, as described in Algorithm 3. AssignFeasible considers the set of jobs provided in input and returns only the subset of feasible jobs. In the process, each feasible job is tentatively assigned (but not scheduled) to a machine. If job *j* is feasible on a single machine, it is added to this machine; if job *j* is feasible on both machines, it is added to the machine with minimum completion time $C_j$ (to account for $f_2$). Note that the procedure AssignFeasible is a variant of the worst-fit greedy heuristic for the well-known bin-packing problem (Korte et al. 2012; Johnson 1973).

In the construction procedure, the selection of the next job is done as follows. First (step 3a), we consider the subset $J'_1 \subset J'$ of the $q_1$ (parameter $< n$) jobs with the largest $w_{j}/p_{j}$ ratio, which is a good heuristic rule with respect to objective $f_2$. Second (steps 3b and 3c), we select the subset $J'_2 \subset J'_1$ containing the $q_2$ (parameter $< q_1$) jobs with the smallest completion times $C_j$, as determined in AssignFeasible. For each job $j \in J'_2$ (and its associated machine), we compute the slack time with the due time of the next PM, and we finally select the job $j^{\star }$ with the smallest slack time. The slack time is the time period between the completion time of the last job scheduled in the considered machine and the due starting time of the next PM. The job $j^{\star }$ is then added (as well as a PM before $j^{\star }$, if required) at the end of the schedule of its associated machine. Note that we choose to schedule jobs with the smallest slack times in order to try to reduce the number of PMs (as we are likely to better use the available working time between two consecutive PMs). Finally, it should be noted that all jobs are considered in the first and second steps when the number of remaining jobs is smaller than $q_1$ and $q_2$, respectively.

Preliminary experiments that are not reported here showed that the following parameter setting is reasonable: $(q_1, q_2) = (0.2n, 0.1n)$. We have tested $q_1 \in \{ 0.05n, 0.1n, 0.2n, 0.3n, 0.4n, 0.5n, n\}$ and $q_2 = \frac{q_1}{2}$. 
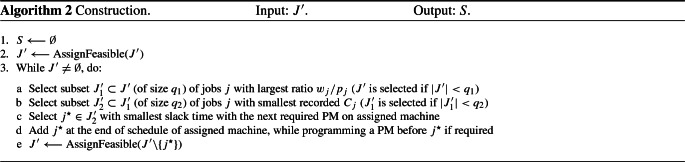

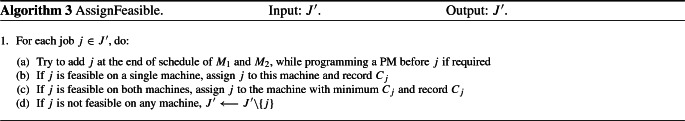


## Local search methods

Introduced in Glover (1989), tabu search is a well-known metaheuristic for solving hard combinatorial optimization problems (Gendreau and Potvin 2019). Starting with some initial solution, a neighborhood of the current solution is generated at each iteration through local modifications (moves). The best solution in the neighborhood then becomes the new current solution, even if it does not provide an improvement. To avoid cycling in the solution space, a tabu list is also defined to forbid certain moves. Since tabu lists are not perfect filters, the tabu status of a move can always be revoked through aspiration criteria if there is no risk of cycling. The tabu search terminates when a stopping criterion is satisfied. The best solution found is returned at the end.

Three local-search methods are proposed sequentially in this section: *Tabu Search with Multiple Neighborhoods* (*TSMN*), *Consistent Tabu Search* (*CTS*) and a *Baseline Local-Search Heuristic* (*BLSH*). *TSMN* and *CTS* are tabu-search metaheuristics, whereas *BLSH* is a simplified version of *CTS* that captures what a decision maker is likely to do in practice. The major difference between *TSMN* and *CTS* relies in the management of feasibility: it is always preserved in *TSMN* but often temporarily violated in *CTS*. More precisely, a move (i.e., a solution modification) is never enforced in *TSMN*: if a move leads to infeasibility, it is ignored (i.e., the move is not investigated further). In contrast, each move is enforced in *CTS*, even if it leads temporarily to an infeasible solution. In such a case, a move is made of two phases: (a) enforce a solution modification; (b) repair the solution to make it feasible again, but without overruling (a). Consequently, considering the same types of moves (e.g., swap, insert), *TSMN* will perform more iterations than *CTS* (i.e., *TSMN* is somewhat faster than *CTS*), as no repair process is employed. The diversification and exploration ability of *TSMN* appears to be better as well, as more solutions are visited per second. However, in counterpart, *CTS* has a better intensification and exploitation ability of the solution space (since many solutions in the same region of the solution space can be investigated).

### Tabu search with multiple neighborhoods *TSMN*

*TSMN* improves the initial starting solution produced by the greedy heuristic *GrH*, while always maintaining feasibility. As shown in Algorithm 4, *TSMN* has three different phases with different neighborhood structures. The algorithm stops when $I^{TSMN }$ global iterations have been performed (step 2) and the best-encountered solution $S^{\star }$ is returned. The latter is updated after each step of Algorithm 4 with respect to the lexicographic ranking $f_1 > f_2$.

Each global iteration corresponds to three consecutive tabu search phases. Phase 1 optimizes objective $f_1$, whereas the sequence of scheduled jobs obtained at the end of Phase 1 is modified in Phases 2 and 3 to optimize $f_2$. In these two last phases, no scheduled job can be rejected; thus, only the sequences of jobs on the two machines are modified.

The neighborhood structures of the tabu search procedures exploit different types of moves for updating the current solution (as explained below). The best non-tabu move—over a random proportion *Pr* of all possible moves—is performed at each iteration of each tabu search procedure. The following values have been tested for parameter *Pr* in our computational study: 0.25, 0.5, 0.75 and 1. Each modification to the current solution needs to be correctly evaluated. It implies that jobs may have to be shifted to the right or to the left (in the latter case, to fill any idle time between two consecutive jobs). However, this is done only from the point of insertion of a new job to the end of the schedule, since nothing changes before the insertion point.

When a move is performed, its reverse move is forbidden for *tab* iterations, where *tab* is an integer randomly chosen in [5, 10] for Phases 1 and 3, and in [3, 7] for Phase 2 (these intervals were tuned after preliminary experiments).*TSMN*comprises a standard criterion aspiration: The tabu status of a move is revoked if it leads to a solution which is better than the best-encountered solution. There is no risk of cycling in this case, since this new best solution has clearly not been previously visited. The stopping criterion for each Phase $l \in \{1, 2, 3\}$ corresponds to a maximum number of iterations, denoted as $I^{TSMN }_l$. Preliminary experiments that are not reported here showed that the following parameter setting is reasonable: $(I^{TSMN }_1, I^{TSMN }_2, I^{TSMN }_3) = (2n, n/5, 3n)$. It should be noted that $I^{TSMN }_2$ is smaller than the two other values given the relatively small size of the corresponding neighborhood, where blocks are moved rather than individual jobs.
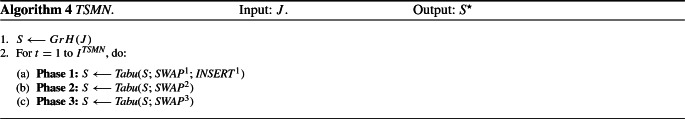

**Fig. 2:**
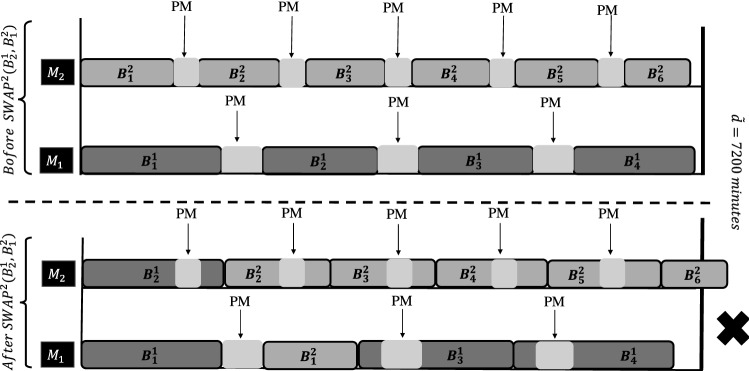
Infeasible solution after swapping two blocks between $M_1$ and $M_2$

The neighborhood structures used in Phases 1, 2 and 3 of *TSMN* are the following:

**Phase 1.** The tabu search *Tabu*(*S*;  *SWAP*$^{1}$; *INSERT*$^{1}$) optimizes only $f_1$ (rejection cost) using a neighborhood structure based on *SWAP*$^{1}$ and *INSERT*$^{1}$. More precisely, a move consists in sequentially swapping two jobs $j \in J_S$ and $j' \in {\overline{J}}_S$ (*SWAP*$^{1}$), and then, in trying to insert in the schedule jobs $j'' \in {\overline{J}}_S$ with a large rejection cost (*INSERT*$^{1}$). In *SWAP*$^{1}$, every pair of jobs $j \in J_S$ and $j' \in {\overline{J}}_S$ are considered for exchange (it could appear as a disadvantage at first sight, but it helps in diversifying the exploration of the solution space). That is, a scheduled job is rejected and replaced by a previously rejected job. After each such potential exchange, the jobs $j'' \in {\overline{J}}_S$ are sorted in decreasing order of rejection cost $u_{j''}$. Then, *INSERT*$^{1}$ considers the jobs in ${\overline{J}}_S$ one by one for insertion in the schedule, with the goal of inserting as many jobs as possible while keeping solution feasibility. Indeed, when a swap is applied between $j \in J_S$ and $j' \in {\overline{J}}_S$, the processing time $p_{j'}$ can well be greater than $p_j$, which may lead to exceeding the deadline ${\tilde{d}}$ (if it occurs, such a swap move is ignored). Conversely, when $p_{j'}$ is smaller than $p_j$, some idle time is created in the schedule and this flexibility can then be exploited by *INSERT*$^{1}$. Note that each swap move is evaluated with respect to objective $f_1$ after having performed the subsequent insertion moves.

**Phase 2.** The tabu searches in Phases 2 and 3 are aimed at improving the scheduling of accepted jobs, as identified in Phase 1, with respect to objective $$f_2$$. The neighborhood structure *SWAP*$$^{2}$$ exchanges every pair of blocks scheduled on the same machine only. Indeed, after some preliminary tests, we discovered that swapping blocks between the two machines was not beneficial because the blocks are not of the same size ($$T_2 < T_1)$$. Consequently, the schedule of machine $$M_2$$ often exceeded the deadline after such a move, as illustrated in Fig. 2 for the exchange of blocks $$B_1^2$$ and $$B_2^1$$.

**Phase 3.** The neighborhood structure in Phase 3 is based on $\hbox {SWAP}^{3}$ moves where pairs of jobs, scheduled on the same machine or not, are exchanged. We consider all possible swaps between two jobs *j* and $j'$, except when *j* appears before $j'$ in the schedule of a given machine and $w_{j'}/p_{j'} < w_j/p_j$ (to be in line with the WSPT rule). The goal here is to obtain a better scheduling of the jobs within the blocks with respect to objective $f_2$.

### Consistent tabu search *CTS*

This tabu search is inspired from the work in Zufferey and Vasquez (2015), where satellite range scheduling problems are addressed. As opposed to *TSMN*, infeasible neighbor solutions are considered but are immediately repaired to restore feasibility. This approach leads to the design of a simpler algorithmic scheme, as shown in Algorithm 5. There are two main phases in *CTS*, each based on a tabu search which is aimed at optimizing one of the two objectives.

An initial solution *S* is first generated using the greedy heuristic *GrH* (step 1). A total number of $I^{CTS }$ global iterations are then performed (step 2). First, objective function $f_1$ is optimized in Phase 1 through insertion moves (based on the below-described *INSERT*$^{2}$ neighborhood structure). A maximum number of $I^{CTS }_1$ iterations are performed with this tabu search, but the procedure is repeated as long as an improvement to the best-encountered solution is observed (step 2a). Next, $f_2$ is optimized in Phase 2 with the *SWAP*$^{4}$ neighborhood structure (step 2b), which is similar to the *SWAP*$^{3}$ used in *TSMN*. This tabu search is stopped after a maximum number of $I^{CTS }_2$ iterations. As opposed to Phase 1, the procedure is not repeated as long as an improvement to the best-encountered solution is observed, because $f_2$ is a secondary objective. Since the two neighborhood structures explored in *CTS* are similar to the ones in Phases 1 and 3 of *TSMN*, we have also fixed $(I^{CTS }_1, I^{CTS }_2) = (2n, 3n)$.

Like *TSMN*, a tabu tenure is associated with each move. In Phase 1, a rejected job is tabu for reinsertion in the schedule for *tab* iterations, whereas the reverse swap move is tabu in Phase 2. In both cases, *tab* is an integer randomly chosen in the interval [5, 10], based on preliminary experiments. The aspiration criterion is the same as in *TSMN*. The neighborhood structures are now presented.

**Phase 1.** Objective $f_1$ (rejection cost) is optimized using the neighborhood structure *INSERT*$^{2}$, where every rejected job is considered for insertion at every position in the schedule of machines $M_1$ and $M_2$. It is important to note that the insertion is enforced even if the deadline ${\tilde{d}}$ is exceeded. In such a case, the tentative solution is immediately repaired by removing accepted jobs that are positioned from the maintenance occurring just before *j* (or from the first job if there is no maintenance before *j*) to the end of the schedule (the selection of such candidate jobs to be removed limits the impact of a job removal on the solution structure, while facilitating the evaluation). More precisely, while the solution is not feasible, we sequentially remove a job $j'$ from $J_S$ in increasing order of their rejection costs (i.e., focus on $f_1$), and we break ties with the smallest ratio $w_{j'}/p_{j'}$ (i.e., focus on $f_2$).

**Phase 2.** Objective $f_2$ (weighted sum of completion times) is optimized using the neighborhood structure *SWAP*$^{4}$ (see *SWAP*$^3$ in *TSMN*). When a swap leads to exceeding the deadline ${\tilde{d}}$ on a machine, feasibility is restored as in Phase 1.
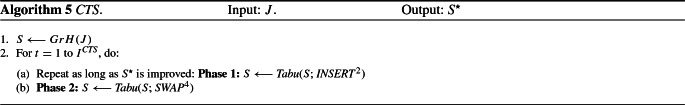


### Baseline local-search heuristic *BLSH*

The baseline local-search heuristic works as *CTS*, with the following simplifications in order to better capture what a decision maker would do in practice.

- Phase (1): perform a local-search descent employing the $INSERT^2$ moves.
- Phase (2): perform a local-search descent employing the $SWAP^4$ moves.

Indeed, from a practical standpoint, a decision maker is likely to perform two modifications. First, s/he could enforce the scheduling of a rejected job *j* (even if other jobs have to be rescheduled or removed to maintain feasibility) because *j* has suddenly received a big priority with respect to a client. Second, s/he could swap two scheduled jobs, for instance, to easily delay the production of a job because its raw material or components are not yet available.

In order to compare, in a fair manner, *BLSH* with *TSMN* and *CTS*, *BLSH* is restarted with a different initial solution provided by *GrH*, as long as the computation time limit (employed for *TSMN* and *CTS* as well) is not reached, and the best-encountered solution is returned at the end.

## Computational experiments

This section reports computational results obtained with the proposed algorithms. The instances are presented in Sect. 6.1. Regarding *TSMN*, the impact of its Phases 2 and 3 is measured in Sect. 6.2. In Sect. 6.3, *TSMN* and the constructive heuristic *GrH* are compared to the MILP for the small instances. Finally, the local search heuristics (*TSMN*, *CTS* and *BLSH*) are compared on the large instances in Sect. 6.4. Given that $f_1$ (rejection cost) is the main objective and subsumes $f_2$ (weighted sum of completion times), we will sometimes report only the values of $f_1$ in the following results for brevity purposes.

All algorithms were coded in Java and the computational experiments were performed on an i7 Intel Core at 2.50 GHz with 16 GB of RAM. The MIP solver is CPLEX 12.7 (default settings) coupled with Concert Technology for the Java interface. The stopping condition of the MILP is 2 h, whereas it is $T = 2n/10$ min for the local-search algorithms (we use a time limit instead of the global iteration counters $I^{TSMN }$ and $I^{CTS }$, to allow a fair comparison among all the meta/heuristics). Preliminary experiments showed that larger values of *T* do not lead to better results. Moreover, it roughly corresponds to an hour of computation for the largest instances, which is in line with the industrial practice (Respen et al. 2017). Since *TSMN*, *CTS* and *BLSH* are all stochastic algorithms, they are run 10 times on each instance and average results are reported. Note by the way that the relative standard deviation is always smaller than 0.2, which is a robustness indicator.

### Presentation of the instances

Since there are no available benchmark instances in the literature for problem (P), we carried out experiments based on randomly generated data, inspired from a real case in the pharmaceutical industry, as reported in Zufferey et al. (2017). We propose small-sized instances (with $n=20$ jobs) for experiments involving the MILP, and large-sized instances for comparing the meta/heuristics (with $n \ge 100$). The weight $w_j=b_j+h_j$ is distributed in the interval [20, 60]. That is, the priority $b_j$ is randomly selected in the set $\lbrace 10, 20, 30 \rbrace$, whereas $h_j$ is uniformly distributed over the interval [10, 30]. Finally, $u_j$ is uniformly distributed over the interval $[b_jp_j/2, 2b_jp_j]$, since the rejection cost of a job *j* depends on its priority $b_j$ and its processing time $p_j$. All the instances and best results can be found in http://dx.doi.org/10.17632/hbs7pm7yhb.1.

#### Small instances

We propose 30 small instances to measure the performance of different meta/heuristics with respect to the CPLEX solver (which relies on the MILP formulation). We have considered $n = 20$ jobs, which is acceptable to often find optimal solutions with CPLEX. A PM is performed on each machine *i* after a maximum of $T_i$ minutes of use, with $T_1=6400$ and $T_2=4800$. The time required to perform a maintenance is set to $4\%$ of $T_i$, which translates into $\delta _1=250$ min and $\delta _2=200$ min. One maintenance is thus required for each machine with respect to the planning horizon of 7200 min (i.e., one full week). Three groups of 10 instances are proposed, denoted as S1, S2 and S3 (where “S” refers to small). Different distributions of processing times (in minutes) are considered in each group, in order to better measure the impact on the rejected jobs.

Group S1 has its processing times $p_j$ uniformly distributed in the interval [315, 1260]. The average value of $p_j$ is thus 787.5 min. In order to roughly estimate the expected number of rejected jobs, we consider that $p_j = 787.5$ for each job *j*. Thus, 15,750 min of work are required to perform 20 jobs and the PMs have a total duration of 250 + 200 = 450 min. Consequently, 16,200 min of activity is required, but the available time for the two machines is 7200 + 7200 = 14,400 min. The missing time is thus 16,200 − 14,400 = 1800 min, which corresponds to the duration of 1800/787.5 = 2.28 jobs. Therefore, the rejection of 3 jobs is expected. Group S2 has its processing times $p_j$ uniformly distributed in the interval [330, 1320] (the average value is 825 min). We can estimate that 3.1 jobs will be rejected, which corresponds to rejecting 4 jobs. Group S3 has its processing times $p_j$ uniformly distributed in the interval [390, 1560] (the average value is 975 min). Similarly, we can estimate that 5.69 jobs will be rejected, which corresponds to rejecting 6 jobs.

#### Large instances

In order the compare the implemented meta/heuristics, we have generated 90 large instances, considering 9 different sizes and 10 instances per size. More precisely, we first propose to consider $n \in \{100, 200, 300 \}$ jobs. Next, to better measure how many additional jobs are rejected (and the impact on the augmentation of $f_1$) with some slight augmentations of *n*, we propose slightly larger instances for each previous value of *n*. Thus, we have three groups of instances, denoted as L1, L2 and L3 (where “L” refers to large). The instance groups, the different values of *n*, the processing-time intervals (uniform distribution), the maintenance parameters $(T_i, \delta _i)$, and the estimations on the number of rejected jobs (as computed above) are presented in Table 1. When a cell is empty, it means that the same value than the one in the cell above is considered.

**Table 1:** Presentation of the large instances

Group	*n*	Interval for $p_j$	$(T_1, \delta _1)$	$(T_2, \delta _2)$	Rejected jobs (estimations)
L1	100	[60, 240]	(960, 40)	(720, 30)	8
	105				13
	110				18
L2	200	[30, 120]	(480, 20)	(360, 15)	16
	210				26
	220				36
L3	300	[20, 80]	(320, 13)	(240, 9)	23
	315				38
	330				53

### Impact of Phase 2 and Phase 3 in *TSMN*

Considering the large instances, Table 2 reports the impact of Phase 2 and Phase 3 of *TSMN* with respect to the objective functions $f_1$ and $f_2$. At this point, we must remember that Phase 1 is aimed at reducing $f_1$, whereas Phases 2 and 3 both focus on $f_2$. For each value of *n* (which involves 10 instances) and each objective function $f_i$ ($i \in \{1,2\}$), we report the following information: the average value of $f_i$ obtained by *TSMN* (i.e., with all its phases), the augmentation percentage (Gap(%)) of $f_i$ if *TSMN* is performed without Phase 2, and the augmentation percentage of $f_i$ if *TSMN* is performed without Phase 3. The larger the gaps are, the worse the solutions are.

The following observations can be made. First and interestingly, even if Phase 2 and Phase 3 are dedicated to $f_2$ only, they both contribute to the reduction of $f_1$ because all the gaps associated with $f_1$ are positive. In other words, Phase 2 and Phase 3 propose promising solutions to Phase 1, and the collaboration among the phases seems to be efficient. Second, regarding $f_2$ (and often regarding $f_1$), Phase 3 seems to be more important than Phase 2, as the values of “Gap(%) w/o Ph3” are larger than “Gap(%) w/o Ph2.” In other words, when reworking the schedule of one machine at a time, swapping two jobs appears to be more beneficial than swapping two blocks. Finally, when observing $f_1$ for one instance group at a time (i.e., L1, L2 and L3), the following trend appears: the gaps decrease when *n* moves from its smallest value to its largest value (i.e., from $n=100$ to $n=110$ in L1, from $n=200$ to $n=220$ in L2, from $n=300$ to $n=330$ in L3). This can be explained by the fact that more jobs are likely to be rejected if we have more jobs to schedule within the same planning horizon. In other words, the augmentation of *n* has a bigger impact on $f_1$ when compared to $f_2$, since $f_1$ is directly associated with job rejection. Therefore, the importance of Phase 2 and Phase 3 often decreases with the increase of *n*.

**Table 2:** Contribution of each phase of *TSMN* with respect to $f_1$ and $f_2$

Instance		$f_1$			$f_2$		
Group	*n*	Average value	Gap(%) w/o Ph2	Gap(%) w/o Ph3	Average value	Gap(%) w/o Ph2	Gap(%) w/o Ph3
L1	100	14,314	5.42	8.49	9,652,798	11.52	12.3
	105	15,279	9.36	7.95	10,080,315	7.22	10.59
	110	31,102	3.06	4.34	9,455,368	5.64	5.95
L2	200	11,147	3.29	3.22	19,218,401	10.27	11.15
	210	20,438	3.87	2.61	18658,798	11.39	12.54
	220	24,772	1.16	1.14	19,269,283	9.81	14.86
L3	300	10,305	1.95	3.06	29,421,036	7.98	9.3
	315	18,108	1.63	5.27	29,077,599	1.73	3.45
	330	29,417	1.5	1.85	27,628,725	6.75	13.07

### Comparison of *TSMN* with the MILP for small instances ($n=20$)

Table 3 presents the following results for the 30 small instances (labeled from I1 to I30) with respect to $f_1$. First, for the MILP, we indicate either “Optimal” if an optimal solution was found within the allowed 2 h of computing time, or “Feasible” if only a feasible solution was found (i.e., without any proof of optimality). The associated time to find an optimal/feasible solution is also given. The number of rejected jobs is indicated in column OUT(MILP) for the MILP, and OUT(*TSMN*) for *TSMN*. Next, we indicate the gap in percentage between *GrH* and MILP, computed as $100 \times [(GrH - \text{ MILP})/\text{MILP}]$ (for a single run of *GrH*). A positive gap means that MILP is better than *GrH*. Finally, we provide the same information, but for the best tabu search *TSMN* (see the next subsection for a comparison between *TSMN* and *CTS*). Note that no computing time is given for *GrH* and *TSMN*, because such methods can find their best solutions in an order of magnitude of a second.

The following observations can be made.

- Considering 20 jobs seems to meet the limits of CPLEX within 2 h of computation, which is a large computing time for such small instances. Indeed, a constructive heuristic such as *GrH* can find the same objective-function values for 16 instances (for which the gaps are 0), but *GrH* requires only up to a second of computing time.
- The MILP is only able to prove optimality when no job is rejected, which corresponds to 9 out of 30 instances. However, the scope if this study is precisely the situation for which the production capacity is not able to schedule all the jobs.
- *GrH* outperforms the MILP for six instances (namely I5, I11, I22, I24, I27 and I30). However, there are eight instances for which the MILP performs better than *GrH*. For such instances, *TSMN* performs as well as the MILP for I14, I15 and I28, and improves the solution provided by the MILP for I4, I21, I25, I26 and I29.
- Unsurprisingly (see the estimations on the number of rejected jobs presented in Sect. 6.1.1), more jobs are rejected when moving from S1 to S3, as the range of $p_j$ values are shifted to larger values. *TSMN* is able to schedule more jobs than the MILP for 10 instances, and both methods schedule the same number of jobs for the other 20 instances.
- *TSMN* is obviously the best method. Moreover, its superiority over the other methods grows when moving from S1 to S3 (i.e., when the production capacity decreases because the job processing times increase).

Considering the objective function $f_2$ and the same time limits, additional experiments (not reported here) were performed for the nine instances for which the MILP is able to prove optimality on $f_1$. For such experiments, each method was constrained by the fact that no job can be rejected (i.e., $f_1$ cannot be deteriorated). The average results are the following: the MILP was able to generate a feasible solution in 7.4 min (without proving optimality within the allocated 2 h); the MILP outperforms *GrH* by 9.63%; *TSMN* outperforms the MILP by 1.87% (but using less than 5 s to do it). In other words, such experiments are in line with the results on $f_1$.

**Table 3:** Comparison of MILP, *GrH* and *TSMN* for small instances ($n = 20$) on $f_1$

Group	Instance	Interval for $p_j$	MILP	Time (MILP)	OUT(MILP)	OUT(*TSMN*)	Gap(%) for *GrH*	Gap(%) for *TSMN*
S1	I1	[315, 1260]	Optimal	123.03	0	0	0	0
	I2		Optimal	147	0	0	0	0
	I3		Feasible	3895	3	3	0	0
	I4		Feasible	24	6	4	0.68	-18
	I5		Feasible	159	6	3	-9.34	-15.25
	I6		Feasible	5986.2	2	2	0	0
	I7		Feasible	8956	2	2	0	0
	I8		Optimal	6895	0	0	0	0
	I9		Optimal	6489.2	0	0	0	0
	I10		Optimal	4899	0	0	0	0
S2	I11	[330, 1320]	Feasible	236.3	3	3	- 8.03	-8.03
	I12		Feasible	350	4	4	0	0
	I13		Feasible	2398	3	3	0	0
	I14		Feasible	14.2	7	5	9.52	0
	I15		Feasible	10.8	4	4	26.62	0
	I16		Optimal	1296	0	0	0	0
	I17		Optimal	2398.7	0	0	0	0
	I18		Optimal	6580	0	0	0	0
	I19		Optimal	3598.99	0	0	0	0
	I20		Feasible	3.98	5	5	0	0
S3	I21	[390, 1560]	Feasible	4877.06	3	2	70.81	-0.53
	I22		Feasible	43.55	1	1	-3.32	-75.20
	I23		Feasible	1171.2	6	6	0	0
	I24		Feasible	106.3	3	2	-5.35	-80.99
	I25		Feasible	5487.13	5	2	10.28	-74.35
	I26		Feasible	33	5	4	6.97	-1.81
	I27		Feasible	19	4	3	-2.94	-23.46
	I28		Feasible	120	4	4	4.49	0
	I29		Feasible	19	7	4	61.37	- 3.07
	I30		Feasible	4.78	3	1	-7.02	-89.88

**Table 4:** Comparison of *BLSH*, *CTS* and *TSMN* with respect to $f_1$

Instance		*BLSH*	*CTS*		*TSMN*	
Group	*n*	Avg. $f_1$	Improvement (%)	Nb. best values	Improvement (%)	Nb. best values
L1	100	16,711	9.47	2	14.34	9
	105	17,716	10.29	6	13.76	9
	110	32,542	0.02	2	4.42	10
L2	200	11,957	2.45	4	6.77	8
	210	21,710	5.29	8	5.86	10
	220	26,425	2.39	7	6.26	10
L3	300	11,033	3.56	6	6.6	10
	315	19,083	0.6	0	5.11	10
	330	30,392	2.28	9	3.21	10

**Table 5:** Comparison of *BLSH*, *CTS* and *TSMN* with respect to $f_2$

Instance		*BLSH*	*CTS*		*TSMN*	
Group	*n*	Avg. $f_2$	Improvement (%)	Nb. best values	Improvement (%)	Nb. best values
L1	100	10,121,838	6.21	9	4.63	1
	105	10,439,980	6.2	10	3.45	0
	110	9,651,740	3.46	9	2.03	1
L2	200	19,332,230	1.87	10	0.59	0
	210	18,696,767	1.63	10	0.20	0
	220	19,304,129	1.52	10	0.18	0
L3	300	29,471,555	4.76	10	0.17	0
	315	29,166,405	4.83	10	0.30	0
	330	29,181,677	8.66	10	5.32	0

### Comparison of *BLSH*, *CTS* and *TSMN* for large instances ($n \ge 100$)

Tables 4 and 5 compare *BLSH*, *CTS* and *TSMN* with respect to objectives $f_1$ and $f_2$, respectively. For each size *n* (which involves a group of 10 instances), we give the following information.

- The average objective-function value of *BLSH*.
- The average improvement percentage of *CTS* over *BLSH* (computed as $100 \times [(BLSH - CTS )/BLSH ]$). A positive value indicates that *CTS* produced improved solutions when compared to *BLSH*. For instance, for $n=10$, *CTS* improves the results of *BLSH* by 9.47%.
- The number of best values (out of the 10 instances) generated by *CTS* while considering all the methods. For instance, for $n=10$, *CTS* has generated 2 times the best solutions.
- The same information is also provided for *TSMN*. Note that if both *TSMN* and *CTS* have generated the best-solution values for a specific size *n* (out of 10 instances), they are counted in both columns labeled as “Nb. best values.” The summation of these two cells can thus exceed 10.

The following observations can be made.

- As expected, for each group L1, L2 and L3, the average value of $f_1$ increases (often significantly) with the increase of *n*. In contrast, $f_2$ does not vary a lot when *n* increases.
- Both *TSMN* and *CTS* are significantly better than *BLSH*. It means that our tabu-search approaches are better than a baseline heuristic aimed at representing a common rule used in practice. Actually, the sequence of moves performed by *BLSH* is likely to be much longer than the sequence of moves that a decision maker would do in practice. In other words, the improvement percentages of *TSMN* and *CTS* with respect to *BLSH* are likely to represent the worst improvement percentages that our tabu-search metaheuristics can bring to practice.
- *TSMN* outperforms *CTS* with respect to $f_1$. Indeed, *TSMN* proposes larger improvements than *CTS* when compared to *BLSH*. We can also remark this superiority when counting the number of best solutions generated by *TSMN*.
- *CTS* outperforms *TSMN* with respect to $f_2$ and the number of best solutions generated.

Relying on the above observations, the superiority of *TSMN* over *CTS* for objective $f_1$ could be explained by the larger diversification ability of *TSMN* (i.e., its capacity to explore various regions of the solution space). Indeed, in contrast with *CTS*, *TSMN* does not spend any energy in repairing infeasible solutions, but only focuses on the quick generation of feasible solutions. Moreover, its Phase 2 brings some diversity (as full blocks of jobs are swapped). On the contrary, the superiority of *CTS* over *TSMN* for objective $f_2$ could be explained by the larger exploitation ability of *CTS* (i.e., its capacity to intensify the search in a specific region of the solution space). Indeed, relying on an efficient repair process, *CTS* is able to enforce some solution modifications and to deeply investigate a move (indeed, *SWAP*$^{4}$ of *CTS* can enforce some swap moves involving two jobs assigned to the same machine, whereas *SWAP*$^{3}$ of *TSMN* cannot).

The number of rejected jobs (denoted here as *R*) is an important KPI from an industrial perspective. For each instance, a way to estimate *R* is presented in Sect. 6.1.1. Considering the large instances, the average performance of all the solution methods with respect to this KPI is presented in Table 6, along with the estimations. The following observations can be made. First and as expected, for each group of instances, *R* increases with the increase of *n*. Second, in line with the previous results, the methods can be ranked as follows: $$GrH< BLSH {}< CTS {} < TSMN $$. Finally, the gap between the estimated *R* and the average number of rejected jobs provided by *TSMN* is reasonable for the instance groups L1 and L2, but not for L3. This highlights the complexity in estimating *R* for the largest instances.

**Table 6:** Average number of rejected jobs

Group	*n*	Estimation	*GrH*	*BLSH*	*CTS*	*TSMN*
L1	100	8	15.9	13.7	12.2	11.2
	105	13	16.7	14.7	11.8	12.8
	110	18	25.4	23.9	19.7	19.6
L2	200	16	25.3	23.8	22.7	20.1
	210	26	40.4	38.3	34	33.3
	220	36	44.9	42	37.4	36.8
L3	300	23	33.75	32.5	30.75	31.5
	315	38	54	51.6	50.75	49.25
	330	53	77.5	77.5	70.75	67

## Conclusion and perspectives

In this work, we studied a parallel-machine scheduling problem (P) with two different machines over a weekly planning horizon, while considering periodic preventive maintenance. Two objectives were considered and addressed with lexicographic optimization, namely minimization of job rejection cost ($f_1$) and weighted sum of job completion times ($f_2$, which can be seen as an inventory penalty). We first introduced a MILP formulation for the problem. Next, we developed a greedy heuristic and two tabu search-based metaheuristics, denoted *TSMN* and *CTS*. A baseline local-search heuristic was also proposed, aimed at representing a current-practice rule. Computational experiments were performed on randomly generated data, inspired from a real case. They showed that *TSMN* outperforms *CTS* for $f_1$ (since *TSMN* is likely to better explore the solution space than *CTS*), whereas *CTS* did better for $f_2$ (which is explained by its better intensification ability, relying on the enforcement of solution modifications thanks to an efficient repair mechanism).

It is important to notice that both the *TSMN* and *CTS* tabu-search methods are easily generalizable for various job-scheduling contexts. They rely on the following main features: a collection of complementary neighborhood structures, the possibility to generate infeasible solutions for implementing important modifications while being able to repair them efficiently, diversification mechanisms (e.g., reschedule a full block of jobs). Such ingredients are useful to find a good balance between diversification and intensification, which are two key features in the design of solution methods.

Various research avenues are possible for the future. On the one hand, alternative problem-solving methodologies could be explored for problem (P), like the adaptive large neighborhood search (ALNS). On the other hand, an extension of (P) could be studied, where several machines and/or optimization criteria are involved. Finally, a stochastic variant of (P) can be investigated, where random machine breakdowns can occur over time.
